# Prognostic value of systemic immune-inflammation index/albumin ratio for immunotherapy-treated patients receiving opioids

**DOI:** 10.1371/journal.pone.0305119

**Published:** 2024-06-27

**Authors:** Lei Yan, Pan Kang, Chengsong Cao, Bu Jinhui, Liu Yong

**Affiliations:** 1 Xuzhou Clinical School of Xuzhou Medical University, Xuzhou, Jiangsu, China; 2 Department of Oncology, Xuzhou Central Hospital, Xuzhou Clinical School of Xuzhou Medical University, Xuzhou Central Hospital Affiliated to Nanjing University of Chinese Medicine, The Xuzhou School of Clinical Medicine of Nanjing Medical University, Xuzhou Central Hospital Affiliated to Medical School of Southeast University, Xuzhou, Jiangsu, China; Kwame Nkrumah University of Science and Technology, GHANA

## Abstract

**Objective:**

This study evaluated the effect of the systemic immune-inflammation index/albumin ratio (SII/ALB) on the prognosis of immunotherapy-treated patients receiving opioids.

**Methods:**

A retrospective analysis was conducted of 185 immunotherapy-treated patients who received opioids at Xuzhou Central Hospital from 01/09/2021 to 01/09/2023. The results of related clinical data were collected during the week before the cancer patients received immunotherapy. The SII/ALB cut-off value was determined, and the relationship between the SII/ALB and clinical pathological parameters was analyzed using the chi-square test. The effect of the SII/ALB on progression-free survival (PFS) was examined using Kaplan-Meier curves and the Cox proportional hazard model.

**Result:**

The SII/ALB cut-off value was 20.86, and patients were divided into low (SII/ALB ≤ 20.86) and high (SII/ALB > 20.86) SII/ALB groups. Adverse reactions (hazard ratio [HR] = 0.108; 95% confidence interval [CI]: 0.061–0.192, P < 0.001) and the SII/ALB (HR = 0.093; 95% CI: 0.057–0.151, P < 0.001) were independent prognostic factors for PFS. Compared with the high SII/ALB group, the low SII/ALB group had longer PFS after opioid treatment (12.2 vs. 5.2 months, P < 0.001).

**Conclusion:**

The SII/ALB is a potentially important prognostic parameter in immunotherapy-treated patients receiving opioids.

## 1 Introduction

Increased cancer incidence and mortality have imposed a heavy burden on global society [1]. Immunotherapy, specifically immune checkpoint inhibitor (ICI) treatment, has become a significant approach in the care of patients with cancer [2]. Cancer pain is a prevalent symptom among patients with cancer, where up to 90% of patients experience cancer pain, and approximately one-third of patients rate the pain as moderate to severe [3]. Opioids are key to alleviating cancer-related pain [4] and enhance patients’ immune function by reducing pain and directly affecting the immune system [5]. However, opioid users might have a lower response to ICI treatment compared to non-opioid users [6]. Retrospective cohort studies explored the relationship between analgesic use and ICI efficacy in patients with advanced cancer [7–13]. Most evidence indicates that opioids might lead to a poor prognosis in patients undergoing ICI therapy [14, 15]. Opioid use was identified as an independent risk factor in patients with advanced non-small cell lung cancer (NSCLC) who received ICIs, which resulted in shorter median overall survival (OS) [12]. Other studies did not report a significant relationship between non-steroidal anti-inflammatory drugs (NSAIDs) and ICIs regarding OS and progression-free survival (PFS) [16]. Given the narrow therapeutic index and inherent toxicity of anti-cancer drugs [17], drug-drug interactions (DDIs) potentially influence ICI treatment efficacy by pharmacodynamically altering drug absorption, distribution, metabolism, or elimination. The ICI biomarkers currently used in clinical practice, such as PD-L1 expression levels and microsatellite instability, are difficult to evaluate, costly, and might not apply to all patients [18, 19]. Hence, a convenient and effective biomarker should be explored to determine the effect of opioids on ICI-treated patients.

Previous studies suggested that hematological indicators could potentially be used to determine ICI efficacy [20–23]. ICI effectiveness relies on the patient’s immune function, which is influenced by inflammation and nutritional status [24]. Immune, inflammatory, and nutritional statuses are interconnected and associated with tumor prognosis [25], while opioids also influence immunity and inflammation [26]. An elevated systemic immune-inflammation index (SII) predicted OS in ICI-treated patients with cancer [23], where elevated SII indicated a poor prognosis. Similarly, albumin (ALB) reflects a patient’s nutritional status and is an early prognostic marker for ICI monotherapy [27].

The SII/ALB ratio was introduced in 2019 [28] and is a comprehensive measure of the immune, inflammatory, and nutritional statuses of patients with malignant tumors, making it a novel indicator for predicting tumor prognosis. The SII/ALB is considered an independent risk factor that affects the prognosis of patients with lung cancer, where an increased ratio indicates a poor prognosis [28, 29].

Nevertheless, the relationship between SII/ALB and the prognosis of concurrent ICI and opioid use remains unclear. This study evaluated and compared the prognostic value of SII/ALB among 185 immunotherapy-treated patients receiving opioids.

## 2 Data and methods

### 2.1 General information

The medical records of 185 immunotherapy-treated patients who received opioids at Xuzhou Central Hospital from 01/09/2021 to 01/09/2023, were retrospectively analyzed. All patients met the following criteria: (1) age ≥ 18 years, (2) had clear pathological evidence of malignant tumors, (3) had baseline cancer pain, (4) at least one lesion for which impact measurement could be performed according to the Response Evaluation Criteria in Solid Tumors (RECIST) version 1.1. Patients with incomplete information were excluded. This study was conducted in accordance with the Declaration of Helsinki (2013 revision). The Xuzhou Central Hospital Ethics Committee approved this study (Approval Number: XZXY-LK-20230822-0144).

### 2.2 Data collection and definitions

“Baseline cancer pain” referred to tumor-related pain that occurred when immunotherapy was initiated. The patients’ pain intensity was rated on a 0–10 numeric rating scale. The patients’ imaging and blood data were collected within one week before receiving ICI treatment. The SII/ALB was calculated as follows: platelet × neutrophil/lymphocyte (10^9^/L)/ALB (g/dL). Specifically, the SII was calculated as platelets × neutrophils/lymphocytes (10^9^/L). The PFS was determined using RECIST version 1.1, which measures the time from the patient’s first use of ICIs to the first occurrence of disease progression or death due to treatment. Additionally, the secondary endpoints’ overall response rate (ORR) and disease control rate (DCR) were evaluated. The immunotherapy response was assessed using RECIST version 1.1 and categorized as complete response (CR), partial response (PR), stable disease (SD), or progressive disease (PD).

### 2.3 Statistical analysis

The statistical analysis was conducted using SPSS 25.0 and GraphPad Prism 9. The SII/ALB was calculated, and the optimal cut-off value for dividing patients into high and low SII/ALB groups was determined using the survminer R package. The two groups were compared using the chi-square test. The survival curve was generated using the Kaplan-Meier method, and survival differences between the two groups were assessed using the log-rank test. The PFS hazard ratio (HR) and 95% confidence interval (CI) were estimated using Cox regression analysis. The Cox multivariate analysis included variables with P < 0.05 in the univariate analysis. Statistical significance was considered at a threshold of P < 0.05.

## 3 Results

### 3.1 SII/ALB cut-off value and patient characteristics

The patients’ SII/ALB were calculated, and the cut-off value was used to divide the patients into low (SII/ALB ≤ 20.86) and high (SII/ALB > 20.86) groups. The 185 patients comprised 67 women (36.22%) and 118 men (63.78%). The SII/ALB stratification parameters were Eastern Cooperative Oncology Group performance status (ECOG PS) 2, stage IV disease, adverse reactions > Grade 2, neutrophils > 1.8 × 10^9^/L, lymphocytes ≤ 1.1 × 10^9^/L, monocytes > 0.6 g/L, platelets > 100 × 10^9^/L, ALB ≤ 35 g/dL, TNF-α ≤ 4.3 pg/mL, IL-6 > 5.4 pg/mL, and SII ≤ 100 (Tables 1–3).

**Table 1:** Clinicopathological parameters of 185 immunotherapy-treated patients receiving opioids.

Characteristic	Total	Low SII/ALB group	High SII/ALB group	*P*
	N = 185(%)	N = 105(56.76%)	N = 80(43.24%)	
Gender				
Female	67(36.22)	37(35.2)	30(37.5)	0.534
Male	118(63.78)	68(64.8)	50(62.5)	
Age				
≤60	80(43.24)	45(42.9)	35(43.8)	0.811
>60	105(56.76)	60(57.1)	45(56.3)	
ECOG PS				
0–1	119(64.32)	90(85.70)	29(36.3)	***<0*.*001*** ***
2	66(35.68)	15(14.30)	51(63.8)	
Staging				
<IV	78(42.16)	50(47.6)	28(35)	0.002**
IV	107(57.84)	55(52.4)	52(65)	
No of metastatic sites				
≤2	94(50.81)	65(61.9)	29(36.3)	0.607
>2	91(49.19)	40(38.1)	51(63.8)	
Cancer type				
NSCLC	78(42.16)	45(42.9)	33(41.3)	0.669
Melanoma	24(12.97)	15(14.3)	9(11.3)	
Hepatobiliary cancer	23(12.43)	13(12.4)	10(12.5)	
Colorectal cancer	21(11.35)	9(8.6)	12(15)	
Head and neck	27(14.59)	18(17.1)	9(11.3)	
Others	12(6.49)	5(4.8)	7(8.8)	
ICI				
Camrelizumab	88(47.57)	46(43.8)	42(52.5)	0.367
Toripalimab	51(27.57)	33(31.4)	18(22.5)	
Sintilimab	35(18.92)	20(19)	15(18.8)	
Others	11(5.95)	6(5.7)	5(6.3)	
Line				
First	96(51.89)	61(59.6)	35(43.8)	0.626
Non- first	89(48.11)	56(40.4)	45(56.3)	
Treatment regimen				
Monotherapy	84(45.41)	55(52.4)	29(36.3)	***0*.*241***
Combined treatment	101(54.59)	50(47.6)	51(63.8)	

* P<0.05

** P<0.01

***P<0.001

**Table 2:** Clinicopathological parameters of 185 immunotherapy-treated patients receiving opioids.

Characteristic	Total	Low SII/ALB group	High SII/ALB group	*P*
	N = 185(%)	N = 105(56.76%)	N = 80(43.24%)	
Opioid doses (mg/d)				
<30	101(54.59)	66(62.9)	35(43.8)	0.099
≥30	84(45.41)	39(37.1)	45(56.3)	
Adverse reaction				
Grade1-2	145(78.38)	94(89.5)	51(63.8)	0.006
Grade>2	40(21.62)	11(10.5)	29(36.3)	
Neutrophils (x109/L)				
≤1.8	16(8.65)	16(15.2)	0(0)	<*0*.001***
>1.8	169(91.35)	89(84.8)	80(100)	
Lymphocyte (x109/L)				
≤1.1	79(42.7)	35(33.3)	44(55)	***0*.*006*** **
>1.1	106(57.3)	70(66.7)	36(45)	
monocyte (g/L)				
≤0.6	117(63.24)	83(79)	34(42.5)	<*0*.001***
>0.6	68(36.76)	22(21)	46(57.5)	
Hemoglobin (g/L)				
≤110	74(40)	34(32.4)	40(50)	***0*.*001*** **
>110	111(60)	71(67.6)	40(50)	
Blood platelet (x109/L)				
≤100	20(10.81)	19(18.1)	1(1.3)	<*0*.001***
>100	165(89.19)	86(81.9)	79(98.8)	

* P<0.05

** P<0.01

***P<0.001

**Table 3:** Clinicopathological parameters of 185 immunotherapy-treated patients receiving opioids.

Characteristic	Total	Low SII/ALB group	High SII/ALB group	*P*
	N = 185(%)	N = 105(56.76%)	N = 80(43.24%)	
ALB(g/L)				
≤35	42(22.7)	18(17.1)	24(30)	***<0*.*001*** ***
>35	143(77.3)	87(82.9)	56(70)	
TNF-α(pg/mL)				
≤4.3	154(83.24)	84(80)	70(87.5)	***0*.*006*** **
>4.3	31(16.76)	21(20)	10(12.5)	
IL1β(pg/mL)				
≤12.4	108(58.38)	58(55.2)	50(62.5)	0.053
>12.4	77(41.62)	47(44.8)	30(37.5)	
IL5(pg/mL)				
≤3.1	103(55.68)	60(57.1)	43(53.8)	0.400
>3.1	82(44.32)	45(42.9)	37(46.3)	
IL6(pg/mL)				
≤5.4	79(42.7)	53(50.5)	26(32.5)	***<0*.*001*** ***
>5.4	106(57.3)	52(49.5)	54(67.5)	
SII				
≤100	105(56.76)	35(33.3)	38(47.5)	***0*.*003*** **
>100	80(43.24)	70(66.7)	42(52.5)	

* P<0.05

** P<0.01

***P<0.001

### 3.2 Survival curve

**Fig 1:**
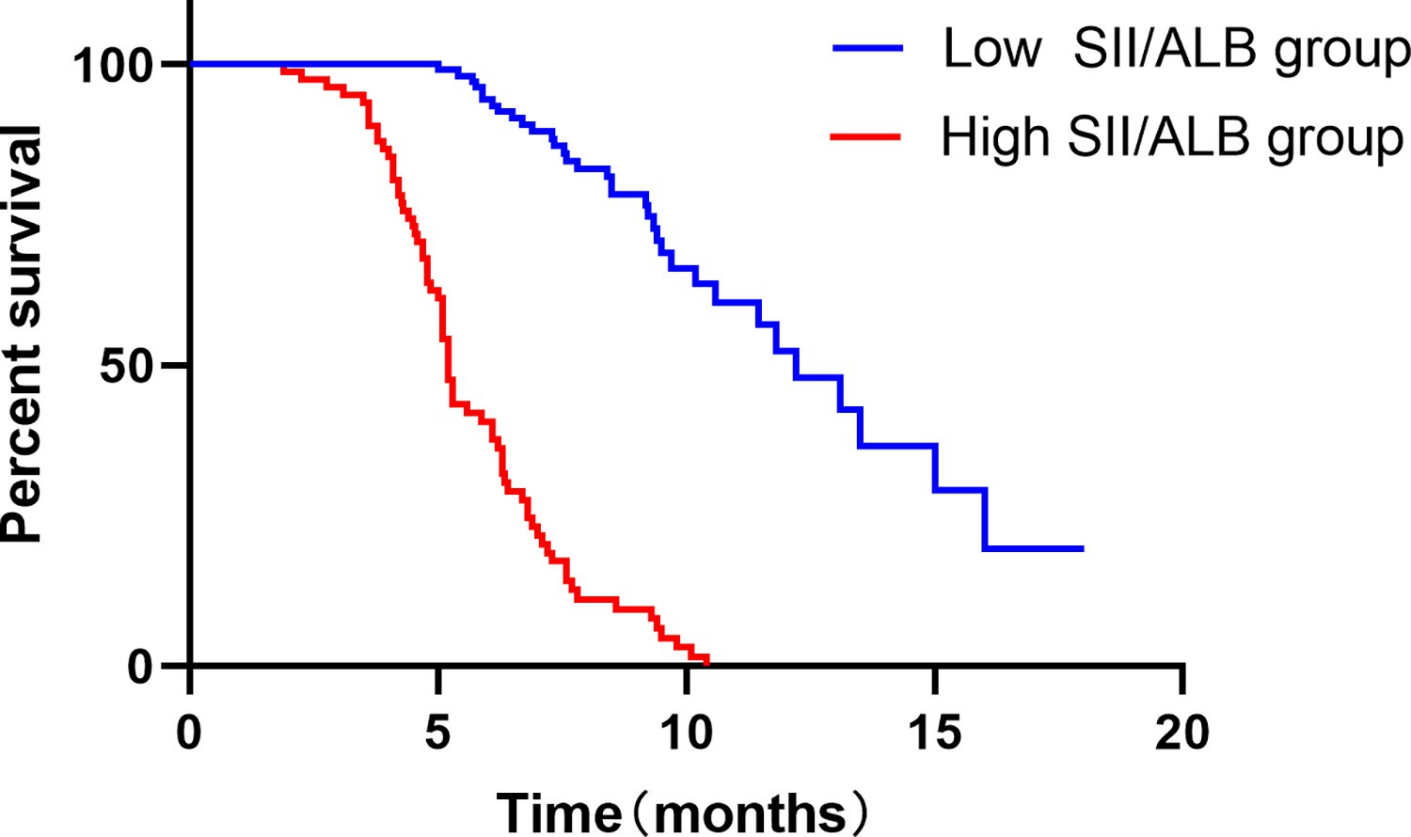
Kaplan-Meier survival curve analysis of the correlation between SII/ALB and PFS.

The correlation between SII/ALB and PFS was analyzed using the Kaplan-Meier survival curve (Fig 1). The Kaplan-Meier survival curve demonstrated that an increased SII/ALB was associated with decreased PFS. The low-SII/ALB group had longer PFS after opioid treatment than the high-SII/ALB group (12.2 vs. 5.2 months, HR = 0.138; 95% CI: 0.8738–0.2172, P < 0.001).

### 3.3 Efficacy evaluation

The chi-square test results revealed that the low-SII/ALB group had higher ORR and DCR than the high-SII/ALB group (ORR = 30.48% vs. 8.75%, P < 0.001; DCR = 66.67% vs. 18.75%, P < 0.001) (Table 4).

**Table 4:** Correlation analysis between SII/ALB and immunotherapy efficacy with opioids.

`	Low SII/ALB group		High SII/ALB group		P
	(n = 105)		(n = 80)		
**CR (%)**	8	7.62%	2	2.50%	
**PR(%)**	24	22.86%	5	6.25%	
**SD(%)**	38	36.19%	8	10.00%	
**PD(%)**	35	33.33%	65	81.25%	
**ORR(%)**	32	30.48%	7	8.75%	***<0*.*001*** ***
**DCR(%)**	70	66.67%	15	18.75%	***<0*.*001*** ***

* P<0.05

** P<0.01

***P<0.001

### 3.4 Univariate and multivariable Cox regression analysis models

The univariate Cox regression analysis results demonstrated that sex, adverse reactions, IL-1β, and SII/ALB were important prognostic indicators of PFS. The multivariable Cox regression analysis demonstrated that adverse reactions (HR = 0.108; 95% CI: 0.061–0.192, P < 0.001) and SII/ALB (HR = 0.093; 95% CI: 0.057–0.151, P < 0.001) were independent prognostic factors of PFS (Tables 5–7).

**Table 5:** Univariate and multivariate Cox proportional hazard regression model survival analysis of SII/ALB for PFS.

Characteristic	Univariate analysis		Multivariate analysis	
	Hazard ratio (95% CI)	*P value*	Hazard ratio (95% CI)	*P value*
Gender				
female	2.033 (1.204–3.432)	*0*.*008*	1.483 (0.989–2.223)	*0*.*057*
male	1			
Age				
≤60	1.295 (0.814–2.06)	0.275		
>60	1			
ECOG PS				
0–1	0.284 (0.077–1.04)	*0*.*057*		
2	1			
Staging				
<Ⅳ	0.233 (0.047–1.162)	0.076		
IV	1			
metastatic number				
≤2	0.296 (0.055–1.604)	0.158		
>2	1			
Cancer type				
NSCLC	0.774 (0.479–1.251)	0.296		
others	1			
ICIs				
PD-1	1.116 (0.443–2.814)	0.815		
others	1			
Line				
1	1.578 (0.573–4.345)	0.378		
≥2	1			
Treatment regimen				
Monotherapy	0.639 (0.096–4.257)	0.644		
Combination	1			

* P<0.05

** P<0.01

***P<0.001

**Table 6:** Univariate and multivariate Cox proportional hazard regression model survival analysis of SII/ALB for PFS.

Characteristic	Univariate analysis		Multivariate analysis	
	Hazard ratio (95% CI)	*P value*	Hazard ratio (95% CI)	*P value*
Opioid doses (mg/d)				
<30	0.814 (0.528–1.255)	0.351		
≥30	1			
Adverse event				
Grade1-2	0.349 (0.163–0.747)	*0*.*007*	0.108 (0.061–0.192)	<*0*.*001*
Grade>2	1			
Neutrophils (x109/L)				
≤1.8	0.761 (0.254–2.281)	0.626		
>1.8	1			
Lymphocyte (x109/L)				
≤1.1	0.678 (0.4–1.148)	0.148		
>1.1	1			
Monocyte (x109/L)				
≤0.6	1.491 (0.894–2.486)	0.126		
>0.6	1			
Hemoglobin (g/L)				
≤110	1.129 (0.688–1.853)	0.631		
>110	1			
Blood platelet (g/L)				
≤100	0.801 (0.296–2.162)	0.661		
>100	1			

* P<0.05 ** P<0.01 ***P<0.001

**Table 7:** Univariate and multivariate Cox proportional hazard regression model survival analysis of SII/ALB for PFS.

Characteristic	Univariate analysis		Multivariate analysis	
	Hazard ratio (95% CI)	*P value*	Hazard ratio (95% CI)	*P value*
ALB (g/L)				
≤35	1.461 (0.838–2.546)	0.191		
>35	1			
TNF-a(pg/mL)				
≤4.3	0.762 (0.386–1.372)	0.400		
>4.3	1			
IL1β(pg/mL)				
≤12.4	0.395 (0.258–0.92)	*0*.*007*	0.725(0.476–1.105)	0.135
>12.4	1			
IL5(pg/mL)				
≤3.1	1.652 (0.848–2.778)	0.105		
>3.1	1			
IL6(pg/mL)				
≤5.4	0.734 (0.438–1.195)	0.218		
>5.4	1			
SII				
≤100	0.645 (0.402–1.113)	0.092		
>100	1			
SII/ALB				
≤20.86	0.037 (0.041–0.175)	<*0*.*001*	0.093(0.057–0.151)	<*0*.*001*
>20.86	1		1	

* P<0.05 ** P<0.01 ***P<0.001

## 4 Discussion

Our findings suggested that SII/ALB and adverse reactions are independent prognostic factors in immunotherapy-treated patients with cancer receiving opioids for pain management. Patients with high SII/ALB and severe adverse reactions had a poorer prognosis. These results agreed with previous studies [14, 15], indicating that patients with cancer with low SII/ALB are more likely to benefit from immunotherapy. This study analyzed data from 185 immunotherapy-treated patients with cancer who received opioids for pain management to assess the prognostic significance of the combined immune, nutritional, and inflammatory markers.

Cancer pain has immunosuppressive effects [30, 31]. Clinical studies have demonstrated that cancer pain suppresses immune function by reducing natural killer (NK) cell activity [32]. ICI efficacy relies on normal immune function, where ICIs inhibit receptors such as PD-1, PD-L1, and CTLA-4 on tumor cells, enhancing the ability of immune cells to attack tumors [33]. Nutrition and inflammatory status also affect the patient’s immune system, influencing its cytotoxicity towards tumors and immunotherapy efficacy [34, 35]. Malnutrition hampers immune cell growth and function, weakening the immune response to tumors. For example, insufficient protein and energy intake reduced T and B cell numbers and activity and decreased macrophage phagocytic function, weakening the body’s tumor-fighting ability [36]. Furthermore, pain induces inflammation, which detrimentally affects the immune system [37]. Continuous immune cell and inflammatory response activation depletes the body’s nutrient reserves, suppressing the immune response to tumors and enhancing tumor escape mechanisms [38]. Opioids alleviate pain and reduce inflammation, improving immunotherapy efficacy. Lastly, the body’s ALB level reflects the patient’s nutritional status and indicates the patient’s liver function reserve and treatment tolerance [39, 40]. Therefore, considering systemic inflammation and ALB levels aids understanding of the immune response.

The hallmarks of cancer are tumor inflammation promotion and immune destruction evasion [41]. The mechanism by which SII/ALB might affect prognostic factors can be discussed from the following perspectives: First, neutrophils promote tumor development by directly interacting with tumor cells or indirectly remodeling the tumor microenvironment [42]. Additionally, neutrophils release pro-inflammatory cytokines and chemokines in the tumor microenvironment, fostering tumor growth and metastasis [43] Neutrophils also release vascular endothelial growth factor (VEGF) and proteases to facilitate angiogenesis and tumor progression [44]. Second, platelets recruit neutrophils and contribute to tumor development [45]. Furthermore, the platelet count is an additional indicator of the systemic inflammatory response and potential microvascular thrombosis and has been associated with angiogenesis through the cytokine VEGF, promoting tumor growth. After platelet activation, VEGF regulates immune and hematopoietic cell migration to the tumor site, intensifying cancer-related inflammation [46]. Third, lymphocytes, the primary cellular component of the human immune system, are involved in anti-cancer immune responses [47]. Specifically, T lymphocytes are crucial for recognizing and eliminating tumor cells, inhibiting their proliferation and migration. Lymphocytes also release cytokines such as TNF and interferon-gamma to impede cancer cell development [48]. Consequently, depleting lymphocytes impairs host anti-tumor activity. Briefly, platelets, neutrophils, and lymphocytes profoundly affect tumor biological behavior [44] by accumulating in blood vessels and releasing VEGF, transforming growth factor beta and platelet-derived growth factor, which promote tumor growth. Fourth, ALB represents the body’s nutritional status and immune defense capability [49], which moderately influences the immune response. Low ALB levels indicate impaired immune function. An increased SII/ALB signifies a relative elevation in platelets and neutrophils and a relative decrease in lymphocytes and ALB. Thus, an elevated SII/ALB might indicate a heightened ability to promote tumors and therefore a poorer prognosis.

This study has several limitations. Firstly, the effects of different opioid types were not different. Thus, multi-center prospective studies are required to confirm whether the opioid type affects the predictive role of SII/ALB. Secondly, the patients’ intestinal microbiota were not examined. The intestinal microbiota is affected by diet and obesity, and this study did not record these factors. Therefore, it was impossible to directly confirm whether dysbiosis is induced in patients taking two drugs simultaneously. Thirdly, data on several confounding factors of comorbidity in the general population were lacking (indicated by the Charlson comorbidity index). Additionally, the melanoma population in the present study lacked information on cancer-specific prognostic factors, such as the PD-L1 tumor proportion score, tumor mutation burden, Breslow thickness, or Clark index. Lastly, As a small-sample, single-center retrospective study, the included population may be heterogeneous. In addition, a total of five types of predominant cancers (NSCLC, melanoma, hepatobiliary cancer, colorectal cancer, and head and neck cancer) were collected in this study and we observed that the SII/ALB ratio demonstrated prognostic relevance across these cancers. However, these findings should not be generalized to the majority of cancer patients receiving opioid-based immunotherapy. This is due to the varying responses of different cancers to ICI. For example, NSCLC and melanoma could show good response to the ICI, but colorectal cancer responded less to the ICI. When these different tumors are combined and analyzed together, this different response may bias the results. Therefore, the correlation analysis in this study relies on sample characteristics and is not representative of the majority of immunotherapy patients receiving opioids.

To the best of our knowledge, this is the first study that predicts the prognosis of cancer patients receiving opioids during their immunotherapy based on hematological indicators. Prior research has largely focused on the outcomes of immunotherapy alone, with minimal attention given to patients experiencing cancer pain managed with opioids. This study serves as an initial exploration into guidance for opioid usage in patients undergoing immunotherapy. Following this research, there are plans to expand investigations into NSCLC and melanoma, increasing the sample size to mitigate variability in response to ICI. In the future, greater attention and research are necessary for immunotherapy patients receiving opioids. The hematological predictors proposed in this study require further validation through large-scale, multicenter prospective studies to ensure the reliability of the conclusions. Therefore, it is currently inappropriate to assume that the correlations observed in this study are universally applicable to all immunotherapy patients treated with opioids.

## 5 Conclusion

The SII/ALB is a potentially important prognostic parameter in immunotherapy-treated patients receiving opioids.

## Supporting information

S1 Data (XLSX)
